# Supplementary material for: Influence of body position on microcirculatory and autonomic responses during arterial occlusion in healthy adults
Source: PLoS One. 2025 Nov 6;20(11):e0335931. doi: 10.1371/journal.pone.0335931 (PMC12591480; doi:10.1371/journal.pone.0335931)
Supplement: Attachment — Submitted filename: LettertoJovenSerdoncillo (1).docx [file pone.0335931.s001.docx]

Dear Editor Joven Serdoncillo

Thank you for your message.

As you can see, we are unable to provide further details beyond what has already been submitted (wich are perfectly aligned with your repeated requests). **Over the last three rounds, we have included both the original and English-translated ethical declarations, ethical protocols, and trial registration documents. In fact, the trial registration is also publicly available online.**

At this stage, we are unclear about your request for additional translation, as the document titled **"Ethical More"** contains all the relevant materials (merged). We would kindly ask you to clarify what specific information you believe is missing. From our perspective, all ethical documentation and procedures have been provided and fully comply with accepted ethical standards and good practices.

We appreciate your guidance and look forward to your clarification.
