# Supplementary material for: Influence of body position on microcirculatory and autonomic responses during arterial occlusion in healthy adults
Source: PLoS One. 2025 Nov 6;20(11):e0335931. doi: 10.1371/journal.pone.0335931 (PMC12591480; doi:10.1371/journal.pone.0335931)
Supplement: Attachment — Submitted filename: reviewersletter.docx [file pone.0335931.s002.docx]

Reviewer #1:

The authors recruited 20 healthy adults to evaluate how body position influences microcirculatory and autonomic responses. The results showed that body position influences both microcirculatory dynamics and autonomic nervous system responses.

AUTHORS: Dear reviewer, thank you so much for your comment.

1. Randomization was implemented for the order of position for each participant. Considering with limited sample size in this study, it would be helpful to also report the actual order at the end.

AUTHORS: Dear Reviewer, thank you very much. We have added the following information to Section 2.5: “To control for potential order effects and ensure balanced exposure, the sequence of positions was randomly assigned using sealed opaque envelopes. Specifically, 5 participants completed supine first, followed by seated and then standing; 4 participants began with seated, then standing, and finally supine; and 6 participants started with standing, followed by supine, and then seated.”

2. Inconsistency in the sample size. The power analysis reported 12, the statistical methods section reported 16, but the results section reported 15. Clarification on these inconsistencies is needed.

AUTHORS: Dear reviewer, thank you so much. We have corrected the typos since we collected data from 15 participants (9 men and 6 women). The information has been updated in both the abstract and the statistical methods section. Regarding the power analysis, it suggested a sample size of 12, but we collected data from more participants to account for potential dropouts, which ultimately did not occur.

3. A nonparametric test was conducted due to the potential non-normality. However, the mean and standard deviation were reported in Table 1. It’s more common and appropriate to report median and IQR instead, while using a nonparametric test.

AUTHORS: Dear Reviewer, thank you very much. We have updated the table 1 and added dedicated rows for the median and IQR to better clarify the information for readers, as you recommended.

4. TR has an extremely large SD as shown in Table 1. This should be further examined, and a sensitivity analysis is warranted. Also, along this concern, it may be informative to add each data observation into the figures to show the variability of observations.

AUTHORS: We thank the reviewer for pointing out the high variability in TR. Sensitivity analysis using Tukey’s method identified one extreme outlier (104 min) in the standing condition. Excluding this outlier, the TR median was 11.90 [10.40–12.68] compared to 12.10 [10.40–12.85] with the full dataset. The overall conclusions remain unchanged: standing shows longer TR than sitting and horizontal, even when the extreme outlier is removed.

Reviewer #2: The quality of this manuscript needs to be improved.

AUTHORS: Dear Reviewer, thank you very much. We have made our best efforts to improve the quality of the manuscript, carefully addressing all comments and suggestions provided. We remain open to further improvements to ensure the highest possible quality.

1. What are the data for quantifying HRV?

AUTHORS: Dear reviewer, thank you so much. We have expanded point 2 of the section 2.6., as follows “HRV was recorded continuously using a Polar H10 chest strap (Polar Electro Oy, Finland) and stored via the HRV Logger application. R-R intervals were sampled at 1000 Hz and exported in raw format. Each participant underwent a standardized 5-minute recording in the designated body position (supine, seated, standing) following a 10-minute stabilization period. HRV serves as a non-invasive indicator of autonomic nervous system function, reflecting the balance between the sympathetic and parasympathetic nervous systems' influences on heart rate [21]. Body position can notably shift this balance, affecting HRV measurements. Studies suggest that HRV typically increases with parasympathetic activity in the supine position, contrasting with a more pronounced sympathetic response when upright. This has relevance in blood flow restriction training (BFRT) and its effects on hyperemic rebound [22]. Frequency-domain indices were calculated using Fast Fourier Transform (FFT) spectral analysis with Welch’s method (window length: 256 s, 50% overlap, Hanning window). All HRV indices were computed using Kubios HRV Premium (Kubios Oy, Finland), which is widely used for clinical and sports applications. The primary outcomes analyzed across positions were median [IQR] values for AVNN (mean of all normal-to-normal R-R intervals, representing overall heart period), SDNN (standard deviation of all NN intervals, reflecting total HRV), LF/HF (ratio of low-frequency (0.04–0.15 Hz) to high-frequency (0.15–0.40 Hz) power, indicating sympathovagal balance), and HR.”

2. The authors stated that “Studies suggest that HRV typically increases with parasympathetic activity in the supine position, contrasting with a more pronounced sympathetic response when upright”. What does “HRV typically increases” mean?

AUTHORS: Dear reviewer, thank you so much. We have improved as follows: “Studies suggest that specific HRV indices reflecting parasympathetic activity — such as higher AVNN, greater SDNN, and increased high-frequency (HF) power — are more pronounced in the supine position [22,23]. In contrast, upright posture elicits a shift toward sympathetic dominance, reflected in shorter AVNN, reduced HF power, increased LF/HF ratio, and higher heart rate [24].”

3. The description of method of determining the arterial occlusion pressure is unclear.

AUTHORS: We thank the reviewer for noting that our description of arterial occlusion pressure (AOP) measurement was unclear. We have revised the Methods section to provide a clearer step-by-step description “(...) Arterial Occlusion Pressure (AOP) was determined individually for each participant on the dominant leg. A 13-cm wide pneumatic cuff (Riester®, Germany) was positioned proximally at the inguinal crease. Using a 2D Doppler ultrasound probe placed over the posterior tibial and dorsalis pedis arteries, arterial flow was continuously monitored. Cuff inflation began at 0 mmHg and increased in 10 mmHg increments after an initial rise to 100 mmHg. At each step, pressure was held for 30 seconds to allow flow stabilization, while Doppler confirmed vessel patency [11]. The AOP was defined as the cuff pressure at which arterial flow was no longer detectable by Doppler. For analysis, we recorded both the minimal pressure reducing resting flow (AOPmin) and the pressure causing complete occlusion (AOP100). This individualized approach accounts for inter-subject differences in limb circumference, vessel depth, and posture, consistent with published BFR protocols. The study employed a standard 5-minute occlusion test on the dominant leg [29].”

4. Regarding the statistical analyses:
1) the Wilcoxon signed-rank test is not the standard post hoc method for the Friedman test, potentially increasing false-positive risks.

AUTHORS: We appreciate the reviewer’s concern and the opportunity to clarify our approach. Because our design involved within-subject repeated measures, the Wilcoxon signed-rank test is an established and widely accepted post hoc test following the Friedman procedure for pairwise comparisons. This choice is supported in the nonparametric statistics literature for related-samples designs (e.g., Gibbons & Chakraborti, 2010). To directly address the concern regarding potential false-positive risks, we took several additional precautions: (i) Bonferroni correction was applied to all Wilcoxon comparisons, and the adjusted p-values are those reported in the main tables; (ii) We further verified robustness by computing exact Wilcoxon p-values with Holm correction, which provides strong family-wise error control while avoiding reliance on large-sample approximations. These supplementary results confirmed that the overall pattern of significance was unchanged; (iii) Finally, we reported Kendall’s W as a global effect size for each Friedman test, which is robust in small-sample settings and demonstrated consistently large effects across outcomes.

2) With small samples (n=16, 15 or 12?), the normal approximation of Z-values may be inaccurate, casting doubt on the reliability of effect size r

AUTHORS: We thank the reviewer for this valuable observation. We agree that in small samples the normal approximation underlying the Wilcoxon Z statistic may reduce the accuracy of effect size r. To address this, we have revised the Statistical Methods section to clarify that r values are reported as descriptive indicators of magnitude only. In addition, we now report Kendall’s W as a robust global effect size for the Friedman test, which does not rely on the normal approximation and is well suited for small-sample nonparametric designs. Across our outcomes, Kendall’s W values ranged from 0.56 to 1.00, consistently indicating large effects, thereby reinforcing the robustness of our findings despite the modest sample size. These were the updates: “The results of the Friedman test indicated that there were significant differences between conditions in rest flow (χ²(2) = 30.000, p < 0.001, W = 1.00), AOPmin (χ²(2) = 28.526, p < 0.001, W = 0.95), AOP100% (χ²(2) = 29.000, p < 0.001, W = 0.97), biological zero (BZ) (χ²(2) = 30.000, p < 0.001, W = 1.00), reactive hyperemia (RHmax) (χ²(2) = 25.200, p < 0.001, W = 0.84), time to peak (χ²(2) = 25.864, p < 0.001, W = 0.86), recovery time (TR) (χ²(2) = 29.525, p < 0.001, W = 0.98), AVNN (χ²(2) = 23.333, p < 0.001, W = 0.78), SDNN (χ²(2) = 26.533, p < 0.001, W = 0.89), LF/HF ratio (χ²(2) = 29.525, p < 0.001, W = 0.98), and HR (χ²(2) = 16.877, p < 0.001, W = 0.56).”

5. The introduction and discussion sections lack clarity in organization and demonstrate insufficient depth. Addittionally, the reationship between changes in reactive hyperemia and changes in HRV was not discussed.

AUTHORS: Dear reviewer, thank you so much. The following was added: “An observation in our study was that upright postures elicited both enhanced reactive hyperemia (greater RHmax, delayed recovery time and time-to-peak) and autonomic shifts toward sympathetic predominance (shorter AVNN, increased LF/HF, elevated HR). These concurrent changes suggest a plausible physiological connection: sympathetic activation is known to attenuate hyperemic vascular responses, likely by opposing vasodilatory mechanisms. For example, in healthy males, acute sympathoexcitation induced by post-exercise circulatory occlusion markedly reduced vasodilatory response to passive leg movement—a surrogate for reactive [54]. Similarly, sympathetic stimulation has been shown to blunt flow-mediated dilation of conduit arteries [55]. Furthermore, in patients with hypertension, greater sympathetic activation during reactive hyperemia has been associated with lower reactive hyperemia index (RHI) by tonometry [56]. Possibly sympathetic predominance in upright posture may contribute to delayed recovery following arterial occlusion in microvascular beds, aligning with the parallel changes observed in our HRV and RH measures.”

6. For the effects of postures on reactive hyperemia, the authors may refer the following papers. Effect of Durations of Wheelchair Tilt-in-Space and Recline on Skin Perfusion Over the Ischial Tuberosity in People With Spinal Cord Injury, 2013, Archives of Physical Medicine and Rehabilitation Skin blood flow dynamics and its role in pressure ulcers, 2013, Journal of Tissue Viability.

AUTHORS: Dear reviewer, thank you so much. We have used both of them to expand our paragraph as follows: “From a clinical perspective, posture-dependent changes in PORH and vascular conduction are significant, particularly in rehabilitation and preoperative settings. For example, AOP tends to be lower in the supine position, raising concerns about underestimating occlusion thresholds during BFRT [9,16]. Research also suggests that limb position affects PORH magnitude, with horizontally or downward-oriented limbs showing stronger hyperemic responses, likely due to hydrostatic pressure effects [4,17]. Beyond limb positioning, posture and unloading strategies also influence cutaneous microcirculation. In individuals with spinal cord injury, durations of wheelchair tilt-in-space and recline significantly alter skin perfusion over pressure-bearing regions [18]. Likewise, reviews of skin blood flow dynamics emphasize how positional changes contribute to ischemia–reperfusion responses and tissue vulnerability [19].”

Reviewer #3: The authors examined the effects of three body positions (supine, seated, and standing) on post-occlusive reactive hyperemia (PORH) and autonomic nervous system activity in healthy individuals. The results demonstrated that the standing position increased arterial occlusion pressure (AOP) and microcirculatory responses, as well as altered heart rate variability (HRV). However, there are still some issues that might need to be clarified.

AUTHORS: Dear Reviewer, thank you very much for your thoughtful and pedagogical comments. They have greatly helped us improve and enhance the clarity of the current version.

SPECIFIC COMMENT
Abstract
1. Line 34-35: Please define all abbreviations (AVNN, SDNN, LF/HF, and HR) at first mention.

AUTHORS: Dear reviewer, thank you so much. We have updated: “average normal-to-normal interval (AVNN), standard deviation of normal-to-normal intervals (SDNN), low-frequency/high-frequency power ratio (LF/HF), and heart rate (HR)”

2. Line 36: “…sensorsStanding…” should be “…sensors. Standing…”?

AUTHORS: Dear reviewer, thank you so much. We have fixed: “(...) sensors. Standing (…)”

3. Line 38: What do “upright postures” mean? Do upright postures include standing and sitting positions? I recommend making terminology consistent.

AUTHORS: We thank the reviewer for pointing out this ambiguity. We have revised the abstract and the main text to clarify that *upright postures* refer specifically to sitting and standing positions, and we now use this terminology consistently throughout the manuscript.

Introduction
1. Again, I recommend making terminology consistent or clearly defining upright positions.

AUTHORS: Dear reviewer, thank you so much. We have updated in introduction as follows “These physiological shifts are especially relevant in BFRT protocols, which are commonly performed in supine, seated, or standing positions. In the present study, we refer to seated and standing collectively as ‘upright positions,’ to distinguish them from the supine posture.”

2. Line 62: Please define EDHF at first mention.

AUTHORS: Dear reviewer, thank you so much. Updated version: “PORH describes the surge in blood flow following the release of a temporary occlusion, serving as a marker for endothelial health. Its regulation involves multiple mechanisms, including the axonal reflex and endothelium-derived factors such as nitric oxide (NO) and endothelium-derived hyperpolarizing factor (EDHF)”

3. Line 74-79: I recommend elaborating more on HRV and autonomic response to justify the purpose of investigating HRV in the PORH protocol. The relationship between HRV and PORH should be described as well.

AUTHORS: Dear reviewer, thank you so much. The following was added: “HRV provides a non-invasive index of ANS balance, reflecting the dynamic interplay between sympathetic and parasympathetic inputs to the heart [20]. Given that the ANS also regulates vascular tone, particularly through sympathetic vasoconstrictor activity, HRV measures can provide indirect insight into microvascular responses such as PORH [21]. Previous work indicates that autonomic shifts influence endothelial function and cutaneous vasodilation, with sympathetic activation attenuating hyperemic responses and parasympathetic predominance facilitating more efficient recovery [22]. Therefore, evaluating HRV in parallel with PORH allows for a more comprehensive assessment of how posture influences both vascular and autonomic components of circulatory regulation.”

4. Line 82: “raising concerns about understanding…” Please clarify what concerns and why the results of this study are critical for optimizing Blood Flow Restriction protocols.

AUTHORS: Dear reviewer, thank you so much. This is the updated version: “AOP tends to be lower in the supine position, which may result in underestimation of the occlusion threshold if these values are later applied during upright exercise [9,16]. Such miscalibration can compromise BFRT efficacy (by providing insufficient stimulus) or increase cardiovascular risk (if pressures are inappropriately elevated). Clarifying these posture-related effects is therefore critical for optimizing BFRT protocols and ensuring both safety and effectiveness [9,16].”

5. Line 91-97: It might not be necessary to describe the detailed outcome measures here. The authors might consider replacing this part with HRV measures and describing detailed outcome measures in the method section.

AUTHORS: Dear reviewer, thank you so much. We have simplified: “By analyzing microcirculatory responses—arterial occlusion pressure (AOP), resting flow (RF), time to peak (TP), recovery time (TR), and biological zero (BZ)—together with autonomic activity assessed via heart rate variability (HRV) parameters [average NN interval (AVNN), standard deviation of NN intervals (SDNN), and low-frequency/high-frequency ratio (LF/HF), this study aims to determine how lower limb positions (supine, sitting, and standing) influence microcirculatory hemodynamic and autonomic responses.”

Methods
1. Line 134: Please provide a reference for McKay’s participant classification or clearly describe the criteria of level 1 classification.

AUTHORS: Dear reviewer, thank you so much. Updated version: “McKay’s participant classification scheme [25];”

2. Line 135: The normal range of ABI should be 0.9-1.4.

AUTHORS: Dear reviewer, thank you so much. Updated version “ankle-brachial index (ABI) within the range of 0.9 and 1.4”

3. Line 140: Why did the study choose 95 mmHg as the diastolic blood pressure threshold? The criteria of hypertension are 140/90.

AUTHORS: We thank the reviewer for this important observation. We have clarified in the Methods that we applied a conservative diastolic blood pressure threshold (≥95 mmHg) to exclude participants, in order to minimize cardiovascular risk during occlusion testing. We also acknowledge in the Discussion that this choice may represent a limitation, as it could restrict generalizability compared to standard hypertension criteria (≥140/90 mmHg) – “Another limitation is the choice of a diastolic blood pressure threshold of 95 mmHg for participant exclusion. Although clinical hypertension is typically defined as ≥140/90 mmHg, we adopted a slightly higher diastolic cut-off to account for measurement variability and to prioritize participant safety during arterial occlusion procedures. This conservative approach may have excluded some individuals with borderline but clinically normal diastolic values, potentially limiting generalizability.”

4. Line 171: Please provide the model of ultrasound and the type of transducer.

AUTHORS: Dear reviewer, thank you so much. Updated: “An experienced physician utilized a SonoScape P20 B-mode ultrasound system (SonoScape, China, 2022) equipped with a linear-array transducer (4–18 MHz) to measure the ankle–brachial index (ABI).”

5. Line 174: What is the model for Doppler ultrasound?

AUTHORS: Dear reviewer, thank you so much. Updated: “A SonoScape P20 B-mode ultrasound system (SonoScape, China, 2022) equipped with a linear-array transducer (4–18 MHz) was used in pulsed Doppler mode, with the probe placed over the posterior tibial and dorsalis pedis arteries to continuously monitor arterial flow.”

6. Line 174: What is the model for the laser Doppler flowmeter?

AUTHORS: Dear reviewer, thank you so much. Updated: “The PeriFlux System 5000 Laser Doppler Flowmeter (Perimed AB, Järfälla, Sweden, 2004) was used to evaluate post-occlusion responses.”

7. Line 203: Again, please define the upright position or simply describe sitting or standing position.

AUTHORS: Dear reviewer, thank you so much. Updated: “In contrast, sitting or standing postures elicits a shift toward sympathetic dominance, reflected in shorter AVNN, reduced HF power, increased LF/HF ratio, and higher heart rate [30].”

8. Line 208: Please describe which artery was measured.

AUTHORS: Dear reviewer, thank you so much. Updated “The dominant leg, determined by self-reported kicking preference, was assessed. Systolic pressures were obtained at the brachial artery and at the ankle using both the posterior tibial and dorsalis pedis arteries. The higher of the two ankle pressures was used for ABI calculation.”

9. Line 212: How did the study determine the dominant leg?

AUTHORS: Dear reviewer, thank you so much. Updated “The dominant leg, determined by self-reported kicking preference, was assessed. Systolic pressures were obtained at the brachial artery and at the ankle using both the posterior tibial and dorsalis pedis arteries. The higher of the two ankle pressures was used for ABI calculation.”

10. Line 213-214: What were the two arteries?

AUTHORS: Dear reviewer, thank you so much. Updated: “The dominant leg, determined by self-reported kicking preference, was assessed. Systolic pressures were obtained at the brachial artery and at the ankle using both the posterior tibial and dorsalis pedis arteries. The higher of the two ankle pressures was used for ABI calculation.”

11. Line 240-243: How did this study determine the value and the time of peak perfusion? How to identify that the resting flow is reduced? The raw LDF signals are noisy. Did you apply any filter or any processing?

AUTHORS: Dear reviewer, thank you so much. Updated: “Raw flux signals were processed offline with a 5-s moving average filter to minimize high-frequency noise. Resting flow (RF) was defined as the mean flux during the 60 s immediately prior to cuff inflation. Peak perfusion (RHmax) was identified as the highest filtered flux value within 2 min of cuff release, and time to peak (TP) was measured as the interval between cuff release and RHmax. Recovery time (TR) was defined as the time required for flux to return to within 5% of baseline RF.”

12. Line 270: Why did this study decide to use the medium effect size to estimate sample size? I suggest using the data from a previous study to perform estimation.

AUTHORS: We thank the reviewer for this comment. At the time of study design, no directly comparable studies had reported effect sizes for PORH and HRV combined across postures. For this reason, we followed Cohen’s widely accepted conventions and selected a medium effect size (f = 0.25) for sample size estimation, ensuring a balance between statistical power and feasibility. We have clarified this in the Methods and noted it as a limitation in the Discussion. Future studies with larger cohorts and pilot data will allow for more precise sample size calculations.

13. Line 275: Although the sequence of conditions is random, the study still needs to evaluate and report the effect of the sequence on outcome measures.

AUTHORS: Dear reviewer, thank you so much. Added: “Exploratory analyses were conducted to examine whether the order in which sessions were performed (first, second, or third) influenced outcome measures. Kruskal–Wallis tests revealed no significant sequence effects for any variable: biological zero (BZ: H(2) = 2.76, p = 0.251), time to peak (TP: H(2) = 1.41, p = 0.494), recovery time (TR: H(2) = 2.89, p = 0.236), AOPmin (H(2) = 0.54, p = 0.765), AOP100 (H(2) = 2.13, p = 0.345), AVNN (H(2) = 2.81, p = 0.245), SDNN (H(2) = 2.85, p = 0.240), LF/HF (H(2) = 2.61, p = 0.271), and HR (H(2) = 0.67, p = 0.716).”

14. Line 285: What test was used for assessing the normality of data?

AUTHORS: Dear reviewer, thank you so much. Added: “Normality of each variable was assessed using the Shapiro–Wilk test (p<0.05).”

Results
1. Line 299: A participant was excluded from the study. Please explain the reason for the dropout.

AUTHORS: Dear reviewer, thank you so much. Fiogure 1 was updated with information “excluded due to elevated resting blood pressure (>95 mmHg diastolic)”

2. Line 321: What is horizontal position? Is it the supine position?

AUTHORS: Dear reviewer, thank you so much. We have updated to supine througout the manuscript.

3. The information in Table 1 is also shown in Figures 4 and 5. The authors might consider removing the figures.

AUTHORS: We thank the reviewer for this suggestion. We believe that presenting both the table and figures adds value for different types of readers. The table provides precise numerical values (medians, IQRs, and effect sizes), which are essential for accurate reporting and reproducibility. The figures, on the other hand, offer an intuitive visual representation of the distribution and variability of the data, facilitating interpretation of posture-related trends at a glance.

Discussion
1. The authors discuss each outcome in each paragraph. It might be better to discuss the effects of positions on PORH and HRV together to better understand the whole physiological changes in different positions more comprehensively.

AUTHORS: Dear reviewer, thank you so much. We have added an integrative paragraph as follows: “Thus, the present findings demonstrate that posture exerts concurrent effects on both microcirculatory and autonomic function. Upright positions (sitting and standing) were characterized by higher resting flow, greater AOP, and prolonged recovery time, alongside a shift toward sympathetic predominance evidenced by shorter AVNN, higher LF/HF ratio, and increased HR. Conversely, the supine position favored lower AOP and stronger parasympathetic activity, consistent with shorter perfusion recovery and reduced vascular resistance. These parallel adaptations suggest that changes in hydrostatic pressure and venous return not only alter local PORH responses but also engage autonomic regulatory mechanisms. Such coupling between vascular and autonomic responses suggest the importance of considering both systems together when evaluating posture effects, particularly for optimizing blood flow restriction training protocols and ensuring cardiovascular safety.”

2. The current discussion lacks physiological explanations for the findings. The authors might need to include some physiological mechanisms of PORH and HRV to interpret the results.

AUTHORS: Dear reviewer, thank you so much. Updates were:“In upright positions, increased hydrostatic pressure in the dependent limb promotes venous pooling and elevates vascular resistance, thereby delaying reperfusion and prolonging recovery time [55]. The release of occlusion induces a surge in shear stress, which activates endothelial vasodilators such as nitric oxide (NO) and endothelium-derived hyperpolarizing factor (EDHF), although this response may be attenuated by sympathetic vasoconstrictor activity [56,57]. In addition, exaggerated central hemodynamic responses in upright positions, including increased stroke volume and sustained heart rate elevation, contribute to greater cardiac output and perfusion pressure, resulting in a more pronounced and sustained hyperemic response compared to the supine position [17].” (...) Mechanistically, the supine position enhances venous return and baroreceptor loading, which sustain vagal outflow and limit sympathetic drive, thereby prolonging AVNN and stabilizing heart rate [31]. In contrast, HR was significantly higher in the standing position, corroborating findings that suggest an increase in sympathetic nervous system activity upon standing [59]. This response reflects baroreceptor unloading during orthostasis, which triggers vagal withdrawal and sympathetic activation to maintain arterial pressure and cerebral perfusion against gravitational stress [60].”

3. The safety of BFR and clinical implications should be discussed.

AUTHORS: Dear reviewer, thank you so much. Added: “From a safety perspective, these findings provide considerations for prescribing BFR exercise. Because AOP was significantly higher in upright positions compared to supine, using cuff pressures determined supine may underestimate actual AOP needed during upright exercise, potentially reducing training efficacy. Conversely, applying high cuff pressures based on supine measurements in upright settings may lead to excessive occlusion, discomfort, or cardiovascular strain. This risk highlights the importance of individualizing AOP in the specific training position. Indeed, recent guidelines recommend applying 40–80% of individual limb occlusion pressure (LOP) to optimize safety and effectiveness in BFR training protocols [68]. Beyond exercise applications, posture-dependent differences in vascular and autonomic responses also bear implications for preoperative assessments and rehabilitation programming. For example, individuals with impaired vascular function—such as the elderly or those with peripheral arterial disease—may exhibit exaggerated hemodynamic and sympathetic reactions to upright posture, which could elevate cardiovascular risk during therapeutic interventions. Monitoring reactive hyperemia and HRV responses may help clinicians adjust BFR protocols or other interventions to individual vascular sensitivity and tolerance levels.”

4. Line 391-404: The authors may minimize repeating the study findings and elaborate more on the physiological mechanisms of how vascular resistance affects AOP.

AUTHORS: Dear reviewer, thank you so much. Updated: “AOP, particularly AOPmin and AOP100%, as well as resting flow (RF), were significantly higher in seated and standing compared to supine positions. These posture-dependent changes are consistent with prior research showing that upright postures require greater cuff pressures to achieve arterial occlusion [43,44]. This effect can be explained by increased hydrostatic pressure in the dependent limb, which elevates transmural pressure and peripheral vascular resistance [45]. When upright, venous pooling reduces venous return and arterial compliance, requiring higher external cuff pressure to compress the vessel lumen. In addition, heightened sympathetic vasoconstrictor tone in upright positions further augments vascular resistance, compounding the increase in AOP [46]. Thus, the interaction of hydrostatic forces, reduced venous return, and autonomic adjustments provides a mechanistic explanation for the higher AOP values observed in upright postures [47,48].”

5. Line 405-415: Again, please elaborate on the physiological mechanisms.

AUTHORS: Dear reviewer, thank you so much. Added: “The greater RH in upright postures can be attributed to a combination of hydrostatic pressure and baroreflex-mediated autonomic adjustments [4]. Elevated hydrostatic pressure in the dependent limb augments the pressure gradient driving reperfusion, while sympathetic activation during orthostasis transiently constrains vasodilation, delaying but also amplifying the hyperemic rebound once occlusion is released [22]. These same mechanisms explain the longer time to peak (TP) and prolonged recovery time (TR) in standing, as increased vascular resistance and delayed clearance of metabolites extend the hyperemic phase.”

6. Line 466-469: “The significant increases in…the supine position.” What clinical recommendation for BFR training do the authors give according to the current results? Is it a good thing to impose greater hemodynamics and autonomic stress during BFR?

**AUTHORS:** Dear reviewer, thank you so much. Updated: “Despite these limitations, our findings have important practical implications. The significant increases in AOP, reactive hyperemia, and sympathetic activity in upright postures suggest that standing and seated positions impose greater hemodynamic and autonomic stress during BFR exercise compared to the supine position. **From a clinical perspective, this dual effect must be interpreted with caution. On one hand, the greater vascular and autonomic load in upright positions may enhance the metabolic and hemodynamic stimulus for training adaptations, which could be advantageous in athletic or rehabilitation contexts. On the other hand, such stress may elevate cardiovascular risk, particularly in individuals with hypertension, peripheral arterial disease, or other vascular impairments. Therefore, we recommend that AOP be measured in the same posture in which BFR exercise is performed, and that lower relative pressures (e.g., 40–50% of AOP) be considered when training in upright positions to balance safety with efficacy. In clinical or frail populations, the supine position may provide a safer option by reducing vascular loading while still delivering the benefits of BFR training. Furthermore, these findings highlight the importance of incorporating posture-specific considerations into vascular health assessments and the design of individualized BFR protocols for populations with compromised circulatory function.“**

7. How do you evaluate the effects of the standing position on BFR training? Any potential harm to individuals with chronic conditions or older adults?

AUTHORS: Dear reviewer, thank you so much. Added “Our results further suggest that while the standing position may provide a stronger hemodynamic and metabolic stimulus—potentially enhancing training adaptations—this also imposes greater autonomic and vascular stress. In healthy or athletic populations, such stress may be tolerable and even desirable for maximizing adaptations. However, in older adults or individuals with chronic conditions such as hypertension, diabetes, or peripheral arterial disease, the additional sympathetic activation and elevated AOP in the standing position could increase cardiovascular risk. For these populations, seated or supine BFR exercise may represent a safer alternative, with more conservative cuff pressures recommended if upright training is pursued.”
